# Norwegian Scabies management after prolonged disease course: A case report

**DOI:** 10.1016/j.ijscr.2019.07.025

**Published:** 2019-07-23

**Authors:** William Aukerman, Karleigh Curfman, Daniel Urias, Kamran Shayesteh

**Affiliations:** Duke LifePoint Conemaugh Memorial Medical Center, Department of General Surgery, USA

**Keywords:** Norwegian Scabies, Scabies, Debridement, Hand

## Abstract

**Introduction:**

Scabies is a well – known, commonly recognized, and frequently diagnosed pathology especially in children, close quarters, living facilities, and immunocompromised patients. An accelerated severe infestation of traditional scabies with limited treatment options is known as the rare entity of Norwegian or Crusted Scabies.

**Case presentation:**

We present the history, clinical manifestations, medical treatments and surgical interventions of a patient with Norwegian Scabies of his hands, which rendered them nonfunctional prior to intervention. The patient was initially misdiagnosed and underwent inappropriate treatments for several years prior to our assessment, and ultimately required surgical intervention that was therapeutic.

**Discussion:**

Norwegian, or Crusted, Scabies is a severe infestation of S. scabiei in which the mite load is extreme in comparison to traditional scabies. This manifests as scaly plaques that are often misdiagnosed for other hyperkeratotic skin lesions. With this misdiagnosis, improper treatments are often recommended, and can even accelerate the manifestation. Traditional scabies therapies can be effective, however often due significant disease progression due to diagnostic delay, invasive measures, such as surgical debridement like presented here, are the only option.

**Conclusion:**

The patient presented in this case harbored a rare infection, known as Norwegian Scabies, for several years, with inappropriate medical therapy. Due to his prolonged inadequate treatments, his disease became so pronounced that the recommended medical treatments were no longer adequate, thus he required surgical debridements which ultimately allowed him to regain function in his hands.

## 1 Introduction

Scabies is a well-established diagnosis and a very common ectoparasitic infection, which has shown trends of infestation in long term care facilities, nursing homes, day care centers, hospitals and other close quarter institutions [1]. Norwegian Scabies is an extremely infectious condition characterized by an extensive infestation of mites [1]. Conventional scabies harbors less than fifteen *S. scabiei* mites and requires fifteen to twenty minutes of close contact for transmission, whereas Norwegian Scabies typically infects immunocompromised and debilitated individuals with tens of thousands of mites [1]. Given the vast mite load, Norwegian Scabies is extremely infectious and results in a significantly increased transmission risk to others within contact [1]. Because of the increased infestation and infection risks, early recognition with appropriate diagnosis and treatment is essential for both therapeutic reasons and infection control [1]]. Typically, topical treatments for conventional scabies, such as benzyl benzoate or permethrin cream, can be utilized to successfully treat Norwegian Scabies [1]. However, for prolonged courses or substantial infestations, surgery may be required as an adjunct therapy in combination with medications to ensure resolution of the infestation and alleviation of symptoms.

The details within this review have been reported in line with the SCARE criteria and documentation reviewed.

## 2 Case report

With this case, we present a 56 – year – old male, without significant medical comorbidities, whose Norwegian Scabies diagnosis was likely misdiagnosed as psoriasis for nearly three years and treated with steroids. The extent of his disease became so advanced that his hands became useless and he ultimately required extensive surgical debulking in addition to medical therapy in order to eradicate his infestation.

The patient was first assessed in the emergency department (ED) for bilateral hand itchiness and bite marks, with concern for Scabies three years prior to our therapeutic surgical debridements. At that time he was provided with Benadryl and permethrin cream; he then followed up with his primary care physician (PCP) who was concerned for psoriasis and instead began treatment with steroids. He had multiple subsequent visits to the ED during the next fourteen months with complaints of worsening hand pain. Over that time, he was treated with antibiotics due to concern for soft tissue infection, continued on steroids for presumed psoriasis, twice was treated with permethrin for suspected Scabies, and at one point was referred to the Plastic Surgery clinic for assessment of his hand lesions. These lesions were described as extensive skin thickening of the volar aspect and white in color, with preserved hand sensation and function, however the etiology remained unclear, so he underwent incisional biopsy of the lesions. Pathology results were significant for atypical verrucous squamous lesions with pseudoepitheliomatous hyperplasia and chronic inflammation of the dermis. The patient was referred, but was unable to follow up with a dermatologist as recommended due to financial constraints.

**Fig. 1:**
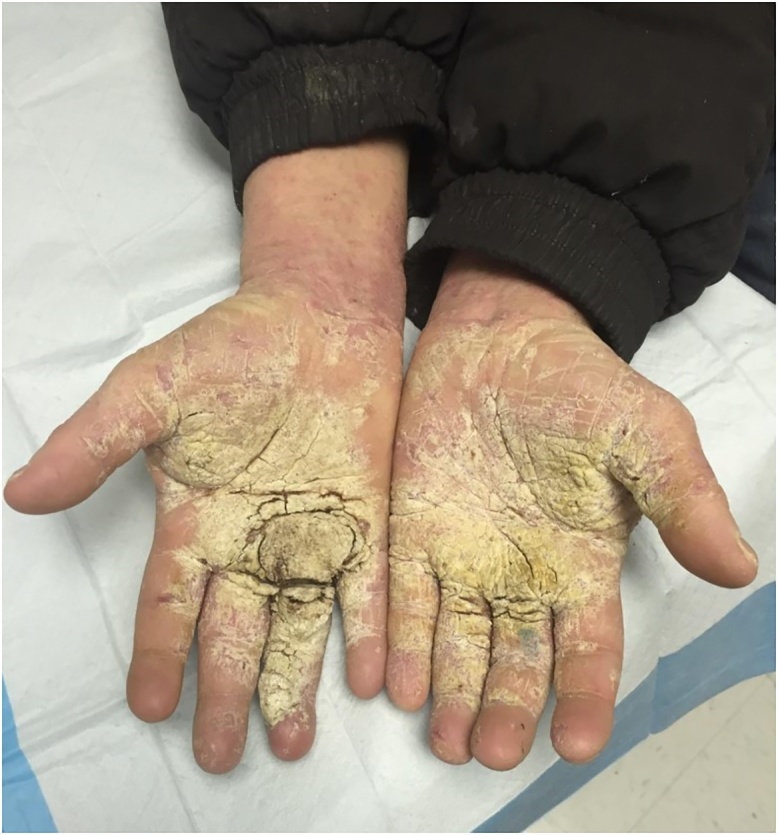
Extensive involvement of bilateral hands with growth and crusts at patient’s pre-operative assessment prior to establishment of Norwegian Scabies diagnosis.

**Fig. 2:**
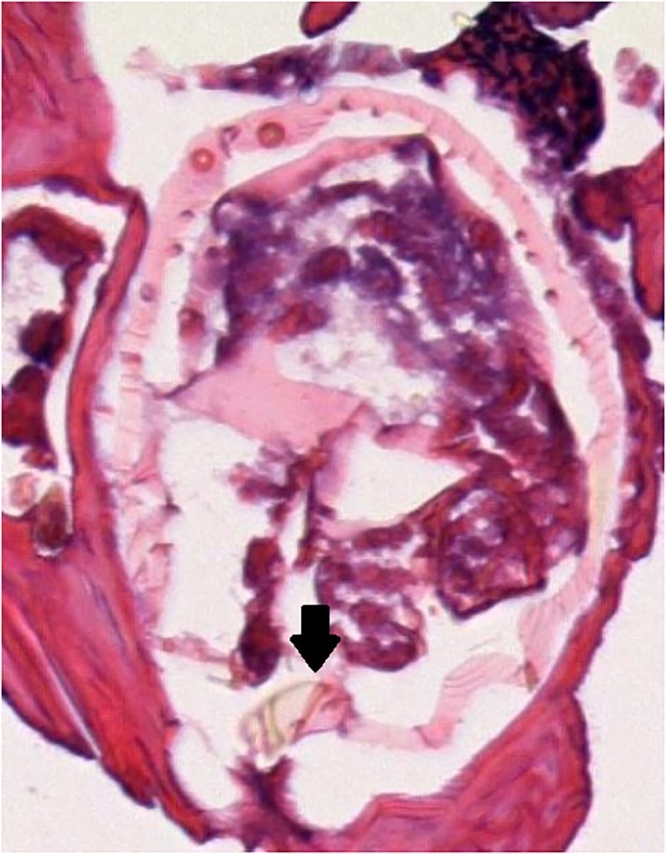
Imaging of initial right hand debridement from pathology report showing cross section of Scabies mite (black arrow), used in confirming patient diagnosis of Norwegian Scabies.

**Fig. 3:**
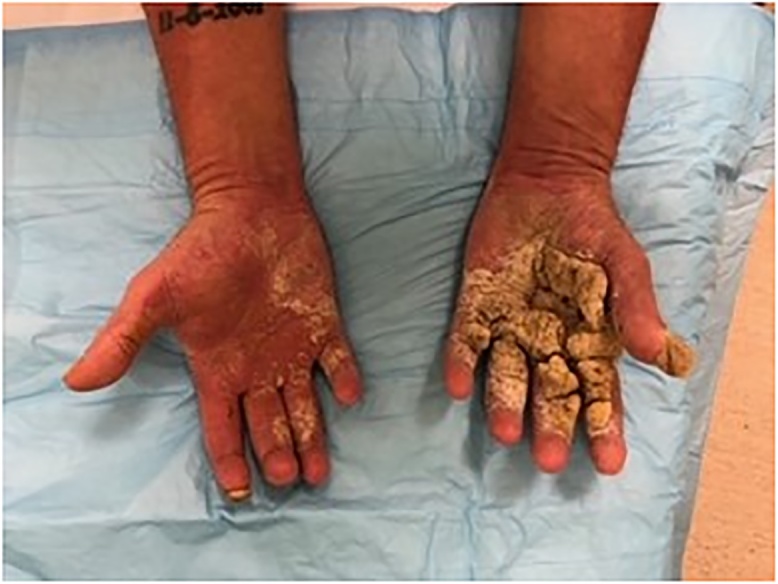
Examination of right hand two months following debridement of crusts and confirmation of Norwegian Scabies diagnosis; left hand, pre debridement, shows extent of disease in comparison.

**Fig. 4:**
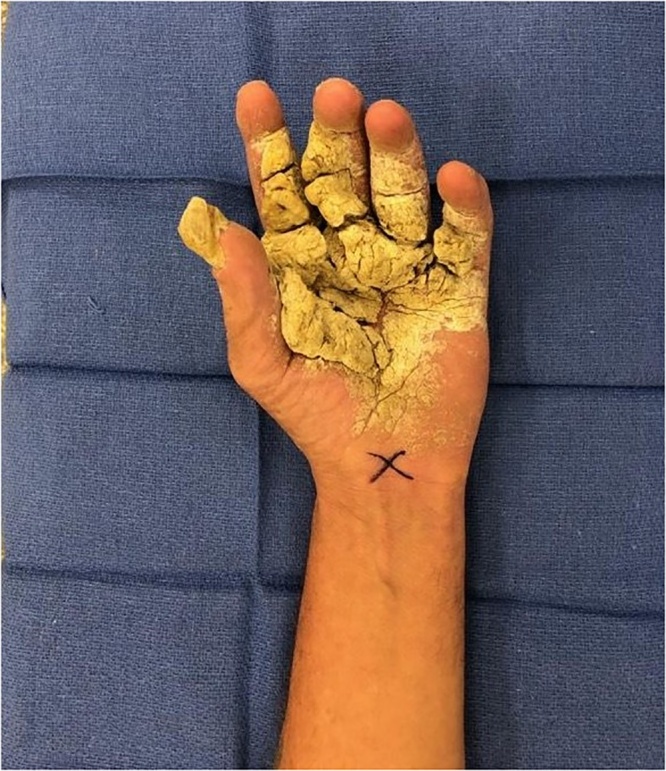
Left hand pre-operatively at time of debridement displaying the significant involvement and infestation of the palmar aspect.

**Fig. 5:**
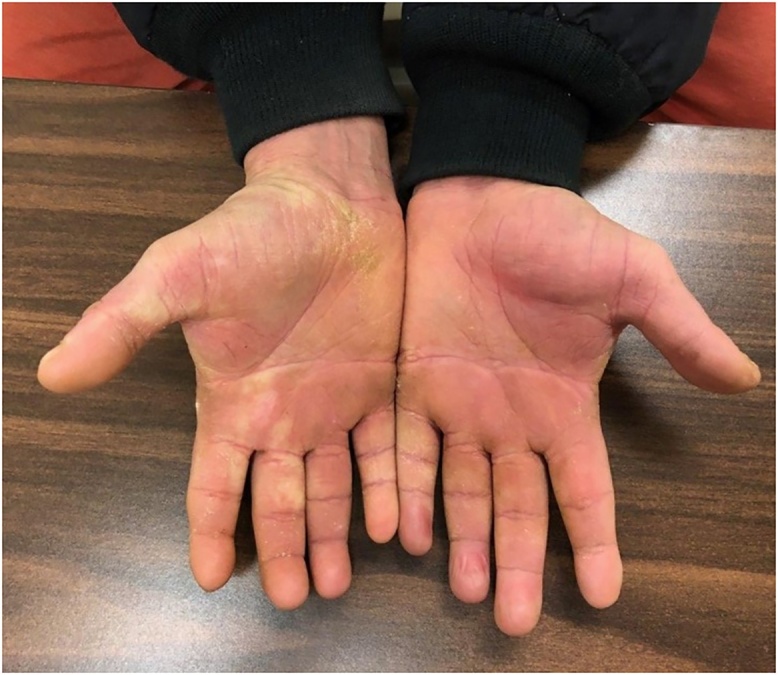
Reassessment at five months from the index right hand debridement and three months from the left hand debridement, showing improved appearance, range of motion, and relief of symptoms.

Several months later, during in inpatient stay for bacteremia, Plastic Surgery was asked to re-evaluate the patient’s hand lesions. His disease had progressed and become so severe that excisional debridement was recommended, however the patient did not follow up with the hand surgeon for another year. Now, three years after his initial assessment, the involvement was so advanced that his hands were essentially nonfunctional due to pain and extensive growth, so he was scheduled for debridement (Fig. 1). He underwent tangential right hand excisional debridement, 12 × 15 cm, for which pathology was positive for Norwegian Scabies infestation (Fig. 2). He was treated with a four week course of medical therapy of both Permethrin cream and Ivermectin. Two months later he was reassessed, and the treatment was repeated for his left hand (Fig. 3, Fig. 4). The patient was seen three months following his left hand debridement, and exhibited resolution of pain, significant increase in range of motion and functionality of his hands, as well as strikingly improved appearance (Fig. 5).

## 3 Discussion

Norwegian Scabies (NS), also known as Crusted Scabies, is a rare skin infestation caused by the parasite *Scarcoptes scabiei*, for which scabies loads can often range from thousands to millions [2]. NS, given its extremely high mite burden within the epidermis, is exceedingly contagious [3]. On presentation, patients often display extensive hyperkeratotic plaques, with yellow – green crusts, most commonly on the torso, extremities, face, and scalp [3]. This described presentation, can often be confused for other diagnoses in those unexperienced or unfamiliar with the parasite and its presentation. Commonly, incorrect pathologies such as pityriasis rosea, tinea versicolor, pediculosis corporis, lichen planus, psoriasis, systemic lupus erythematous, bullous pemphigoid, eczema, or adverse drug reactions are mistakenly diagnosed [1,4]. Risks of misdiagnosis commonly include transmission to others and uncontrolled, bolstering infection, but can also be associated with bacterial infections, mainly *S. aureus* [4]. Many of these differential diagnoses are treated with steroid therapy, which can lead to an atypical scabies presentation known as “Scabies Incognito”, which further complicates treatment by masking the correct diagnosis [5]. In addition, though not witnessed in our patient, Norwegian Scabies has a high occurrence rate in patients who are immunosuppressed [6]. Because of this, the infection should raise suspicion for possible concurrent Human Immunodeficiency Virus (HIV) diagnosis, and confirmatory diagnostic testing must be performed [7]. Per reports, the invasion has been previously associated with multiple other disease types in which nearly half of the patients involved harbored: Human T - lymphotrophic virus – 1, Adult T – Cell Lyphoma, Leprosy, Epidermolysis bullosa, IgA deficiency, Langerhans Cell Histiocytosis, Neurtropenia, Myelodysplasia, pregnancy, as well as HIV [6].

Once confirmation of this elusive diagnosis has been achieved, commencement of appropriate medical therapies should be immediate, due to the high transmission risk. Uncomplicated scabies infestations are often treated with acaricides including: Benzyl benzoate, Crotamiton cream, Lindane, Permethrin cream, Malathion lotion, sulfur petroleum, or Ivermectin [1]. Benzyl benzoate, Permethrin cream, and Malathion lotion have been shown to be very effective against scabies infestations and are reasonable treatment options. Benzyl benzoate is an especially favorable option in adults as it is proven to show excellent therapeutic response, is inexpensive, and treatment often requires only one dose, though a second dose 2–7 days later can be recommended [1]. Though in children, Benzyl benzoate can cause irritation, which is why Malathion lotion or Permethrin cream are often favored [4]. Other recommended treatment options, such as Crotamiton and Lindane can be effective, though they are not without significant drawbacks. Crotamiton cream requires repeated dosing of five times daily, with cure rates reaching only 60%, while Lindane is often not a first choice because of its neurotoxicity risk [1].

Given their effects in uncomplicated scabies infections, traditional treatments have also been applied successfully to the management of Norwegian Scabies, with slight alterations. The primary recognizable difference is that treatments will require multiple dosings. Because of this, Lindane, with its neurotoxic risk, becomes contraindicated [1]. Topical Permethrin cream and intravenous (IV) Ivermectin have both shown promising results in the treatment of NS [1,8]. Permethrin requires weekly applications for at least six weeks to obtain adequate response, while the IV Ivermectin regimen includes an initial dose, followed by subsequent weekly IV doses for at least two to three weeks [1]. In addition to acaricides, keratolytic agents such as salicylic acid or urea, can be used in adjunct to increase destruction of any hyperkeratotic skin lesions and further enhance the penetration of applied topical medications [1]. As success with medical therapy is often achieved, few similar cases have been reported in which the disease burden was so advanced that keratolytic agents were decided against in favor of surgical debridement [9]. In severe infestations, surgical debridement has been used as a successful adjunct procedure in order to debulk the lesions and intensify the ability of topical medications to penetrate and eliminate the mites [9].

## 4 Conclusion

Norwegian Scabies is rare pathology within the realm of Scabies and ectoparasitic infections. The patient presented with harbored ailment for years without knowledge of cause, proper confirmed diagnosis, treatment, or improvement of symptoms. Due to containment of the disease for so many years with lack of any suitable treatments, the extent of his infestation became so pronounced, that an appropriate debulking procedure in combination with medical treatment was his only chance for a cure. Fortunately the patient tolerated his procedures well, had an exceptional cosmetic response, experienced essentially complete resolution of symptoms, and after three long years, eradication of his disease has now been achieved.

## Funding

The authors received no funding in the preparation of this manuscript.

## Ethical approval

Ethical approval for this case report was obtained from Duke LifePoint Conemaugh Memorial Medical Center’s additional review board, ID 18–27.

## Consent

Informed consent was obtained from the patient in the presence of witness, for publication of this case report, "Norwegian Scabies Management after Prolonged Disease Course: A Case Report" and any accompanying images. A copy of the consent obtained is available for review by the editor of this general.

## Author contribution

Contributors to the conception and design, acquisition of data, and interpretation of data: WA, KC, DU.

Manuscript writing and drafting: WA, KC, DU.

Revising it critically for important intellectual content: WA, KC, DU, KS.

Final approval of the version to be published: WA, KC, DU, KS.

## Registration of research studies

NA.

## Guarantor

William D. Aukerman, MD.

## Provenance and peer review

Not commissioned, externally peer-reviewed.

## Declaration of Competing Interest

The authors received no funding and have no additional relationships in the preparation of this manuscript.
